# Capsicum and Quinine

**Published:** 1879-04

**Authors:** 


CAPSICUM AND QUININE.—Capsicum combined with quinine will diminish the size of the dose requisite, and the same may be said of ginger and other aromatics. A good dose of capsicum combined with twenty grains of quinine will act as well as thirty grains of quinine without the capsicum. Spices in general stimulate the portal circulation and promote the flow of bile, and hence their universal use in hot climates. There is a tendency on the part of quinine and capsicum to purge, and sometimes to purge violently. In such cases the purgative action is caused by the increased flow of bile produced by the capsicum. Ginger and quinine when combined do not purge, and it makes a very good combination. If the medicine is administered in form of pills, capsicum may be preferable, because of the less bulk required; but if desirable, the ginger may be given separately, and with the same effect as when combined with quinine. The proportions should be one grain of capsicum to three grains of quinine; with ginger, one grain of each.—*Richmond and Louisville Medical Journal.*

